# An Addendum to Injury Rates in Iranian Taekwondo Athletes; a Prospective Study

**Published:** 2010-06

**Authors:** Willy Pieter, Mohsen Rostami, Vahid Ziaee

**Affiliations:** 1 ExRA, Inc., Ann Arbor, MI, USA; 2 Sports Medicine Research Center, Tehran University of Medical Sciences, IR Iran

**Keywords:** Taekwondo, Martial arts, Sport injury, Combat sports, Athlete exposure

## Abstract

**Purpose:**

The objective of this study was to compare the Iranian *taekwondo-in* statistically in terms of total injury rates to international counterparts as gleaned from the extant literature.

**Methods:**

The Iranian sample consisted of 204 male *taekwondo-in* participating in the national championship. The international sample included the participants in national and international tournaments. Validated standard questionnaires were employed at all tournaments to collect injury data that were always diagnosed by the respective tournament physicians. An injury was defined as any circumstance for which assistance was sought from the medical personnel. In addition to injury rates, 95% confidence intervals (CIs) around the rates were computed. To assess which group was at higher risk, odds ratios were calculated, including the 95%CIs.

**Results:**

Compared to Greek counterparts, the injury rate for the Iranian *taekwondo-in* was statistically significantly higher. The Iranians were also at a higher risk to incur an injury: OR = 11.2 (95%CI: 6.60–18.88, *P*<0.001, CLR = 2.86). When comparing the Iranian *taekwondo-in* to their colleagues competing at the 1999 World Championships, the former recorded a statistically significantly lower injury rate but the latter were not at a higher risk (OR = 0.61, 95%CI: 0.41–0.91, *P*=0.014, CLR = 2.20).

**Conclusion:**

A statistical comparison of total injury rates in Iranian and international *taekwondo-in* revealed no difference between the two groups. However, what is of concern is that the total injury rate across taekwondo studies is significantly higher than those reported for American football.

## INTRODUCTION

Full-contact or Olympic taekwondo is a modern sport from the second half of the 20^th^ century^[1–4]^. Despite its young age, it became an Olympic demonstration sport in 1988 (Seoul) and 1992 (Barcelona) and a full medal contender at the 2000 Sydney Olympic Games. The earliest known epidemiological study on taekwondo injuries was published in 1989 based on the 1988 US Olympic Taekwondo Team Trials^[5]^. A proliferation of (observational) injury research has subsequently ensued on both young and adult taekwondo athletes (*taekwondo-in*) ^[e.g.,6–22]^ with a recent meta-analysis of the available literature to date^[23]^. The most recent of these investigations was reported by Ziaee et al.^[24]^, which is believed to be the first of its kind in Iran.

The risk of participating in any sport is typically expressed in terms of comparing total injury rates. For instance, American adult male elite *taekwondo-in* were reported to incur an injury rate of 127.36 injuries/1,000 athlete-exposures (A-E) (95%CI: 79.33–175.40)^[20]^, Their Canadian colleagues recorded a rate of 79.91/1,000 A-E (95%CI: 53.44–106.38)^[18]^.

Due to the increase of research on the epidemiology of taekwondo competition injuries, it has now become possible to compare injury rates statistically and identify those are at higher risk of injury. The purpose of this study, then, was to add to the existing body of knowledge by comparing the Iranian *taekwondo-in*^[24]^ statistically in terms of total injury rates to international counterparts as gleaned from the extant literature. Recent reviews of taekwondo competition injuries^[3, 25]^ served as the basis for comparing the Iranian athletes to get a firmer grasp of what was reported^[24]^.

## METHODS AND SUBJECTS

As reported by Ziaee et al.^[24]^, the Iranian sample consisted of 204 male *taekwondo-in* participating in the national championship. They competed in bouts lasting 3 rounds of 2 minutes each with 30-second breaks in between rounds. The international sample participated in national and international tournaments in bouts consisting of either 3 rounds of 2 minutes with 30-second breaks in between rounds or 3 rounds of 3 minutes each interspersed with 1-minute breaks in between rounds. The competition rules used at all competitions were those of the World Taekwondo Federation (WTF).

Validated standard questionnaires were employed at all tournaments to collect injury data that were always diagnosed by the respective tournament physicians. An injury was defined as any circumstance for which assistance was sought from the medical personnel.

Exposure data were gathered from records of bouts actually fought. Since two athletes contested each bout, there were two athlete-exposures (A-E) per match. Injury rates were calculated as (number of injuries/athlete-exposures) x 1,000 = number of injuries per 1,000 A-E^[5, 24]^.

In addition to injury rates, 95% confidence intervals (CIs) around the rates were computed. Statistical comparisons were made with one-tournament groups in which elite *taekwondo-in* competed. To this end, the following studies were included: Pieter et al.^[8]^, Beis et al.^[14]^, Koh et al.^[15]^ and Kazemi and Pieter^[18]^.

To assess which group was at higher risk, odds ratios were calculated, including the 95%CIs. The level of significance was set at 0.05.

## RESULTS

Table 1 shows comparative injury rates in elite *taekwondo-in* from various countries. When compared to those competing at the European Cup^[8]^, the Iranian *taekwondo-in* were not at a lower risk of incurring an injury: OR = 0.85 (95%CI: 0.52–1.41, *P* = 0.528, CLR = 2.71), although the injury rate was significantly lower.

**Table 1:** Competition injury rates per 1,000 athlete-exposures in taekwondo athletes

Study	Country	Sample size	Injury rate (95%CI)
**Ziaee et al.**^[24]^	Iran	204	69.51 (55.38–83.64)
**Pieter et al.**^[8]^	Europe*	67	139.54 (93.95–185.12)
**Pieter and Zemper**^[26]^	American	1,665	95.07 (84.72–105.42)
**Koh et al.**^[15]^	World**	330	120.81 (92.91–148.72)
**Beis et al.**^[14]^	Greece	533	20.55 (11.76–29.34)
**Kazemi and Pieter**^[18]^	Canada	219	79.91 (53.44–106.38)

* European Cup

** World Championship

Compared to Greek counterparts^[14]^, the injury rate for the Iranian *taekwondo-in* was statistically significantly higher. The Iranians were also at a higher risk to sustain an injury: OR = 11.2 (95%CI: 6.60–18.88, *P*<0.001, CLR = 2.86).

When comparing the Iranian *taekwondo-in* to their colleagues competing at the 1999 World Championships^[15]^, the former recorded a statistically significantly lower injury rate but the latter were not at a higher risk (OR = 0.61, 95%CI: 0.41–0.91, *P* = 0.014, CLR = 2.20).

There was no difference in injury rate between the Iranian *taekwondo-in* and their Canadian colleagues (Kazemi and Pieter, 2004^[18]^). The Canadians were also not at a higher risk (OR = 0.42, 95%CI: 0.26–0.66, *P*<0.001, CLR = 2.54).

## DISCUSSION

To the best of our knowledge, this is only the second statistical comparison of injury rates in taekwondo between studies from a variety of geographical locations^[3]^, while at risk groups were also recently identified^[25]^. However, it is not clear why the Greek *taekwondo-in* recorded the lowest injury rate. It may be that they were not willing to report an injury^[25]^. Although the elite Greek *taekwondo-in* had a lower point estimate than British recreational counterparts 51.28 (95%CI: 1.02–101.54)^[25]^, this was not statistically significant, which was suggested to be due to the small sample size of the British *taekwondo-in*^[25, 27]^.

On the other hand, a similarly small sample size of American elite *taekwondo-in* yielded an injury rate of 127.4/1,000 A-E (96%CI: (79.3–175.4)^[5]^, which was not statistically significantly different from the rate recorded by the Iranian taekwondo athletes. However, it was significantly larger than that of the Greek colleagues ^[14]^

Although experience was suggested to be related to the occurrence of injuries, this was only reported for time-loss injuries^[25]^. Future research should investigate this relationship in general injuries as well. To put the injury rates of taekwondo into perspective, it is instructive to compare them to those reported for other combat sports (see Table 2).

**Table 2:** Comparative injury rates per 1,000 A-E in American football and combat sports

Sport	Injury rate
**Iranian elite *taekwondo-in***^[24]^	69.51 (55.38–83.64)
**American football**^[28]^	36.11 (35.70–36.52)
**Dutch elite *karateka***^[29]^	168.87 (144.13–193.61)
**Iranian Elite *karateka***^[30]^	186 (159–205)
**British elite *judoka***^[31]^	48.54 (18.45–78.63)
**Asian elite *judoka***^[32]^	25.18 (6.53–43.83)
**Elite *karateka***^[33]^	157.64 (145.20–170.08)
**Wrestling**^[34]^	26.2 (25.2–27.2)

American football and wrestling injuries were collected over a 15-year period, while the other injury rates in Table 2 were all based on one competition only. In a recent review of taekwondo injuries, it was revealed that the rates across studies published from 1989 to 2007 ranged from 20.6 to 139.5 per 1,000 A-E, which is based on mostly elite *taekwondo-in* except for one study on recreationalists ^[25]^. The latter incurred an injury rate of 51.28 (95%CI: 1.02–101.54)^[27]^. The large confidence interval is due to the small sample and cell sizes. The injury rate for the elite *taekwondo-in* only would be 89.40/1,000 A-E (95%CI: 79.98–100.66), which is statistically not different from the rate reported for the Iranian *taekwondo-in*^[24]^. However, it is significantly higher than that of American football as well as wrestling (Table 2).

Since taekwondo competitions are currently contested in bouts of 3 rounds of 2 minutes each and since each athlete might have to be engaged in several bouts before reaching the finals, the chances of incurring an injury over the course of each tournament is a cause for concern for all involved. Research on injuries over time during a competitive match, regardless of the sport, is scarce. For instance, in American and Australian football, injuries were reported to increase later in the match^[35, 36]^.

In combat sports, injuries were reported to increase over time in karate competition^[37]^. In 2001, Beis et al.^[38]^ reported taekwondo injuries in relation to time of competition, while Ziaee et al.^[24]^ suggested that most injuries were sustained in the third round of a match. Fatigue is suggested to be at the basis of this finding^[37, 38]^. Future research should consider taking into account the time of injury occurrence, which in turn may lead to improved preventive measures, such as better conditioning of the athletes^[39, 40]^ or changing competition tactics.

## CONCLUSION

A statistical comparison of total injury rates in Iranian and international *taekwondo-in* revealed no difference between the two groups. However, what is of concern is that the total injury rate across taekwondo studies is significantly higher than those reported for American football. The international governing body for Olympic taekwondo and each national counterpart may want to consider more effective preventive measures.

## References

[1] Pieter W (1981). Etymological notes on the terminology of some Korean martial arts. Asian J Physic Educ.

[2] Capener S (1995). Problems in the identity and philosophy of t'aegwŏndo and their historical causes. Korea J.

[3] Pieter W, Kordi R, Maffulli N, Wroble RR, Wallace AW (2009). Taekwondo. Combat Sports Medicine.

[4] Pieter W (2009). Historical aspects of t'aekwŏndo. Archives of Budo.

[5] Zemper ED, Pieter W (1989). Injury rates during the US Olympic Team Trials for taekwondo. Br J Sports Med.

[6] Oler M, Tomson W, Pepe H (1991). Morbidity and mortality in the martial arts: a warning. J Trauma: Injury, Infection and Critical Care.

[7] Pieter W, Lufting R (1994). Injuries at the 1991 Taekwondo World Championships. J Sports Traumatol Rel Res.

[8] Pieter W, Van Ryssegem G, Lufting R, Heijmans J (1995). Injury situation and injury mechanism at the 1993 European Taekwondo Cup. J Hum Mov Stud.

[9] Pieter W, Zemper ED (1997). Time-loss injuries in Junior Olympic taekwondo athletes. Sports Exerc Inj.

[10] Pieter W (1997). Fractures in karate competition. J Sports Traumatol Rel Res.

[11] Pieter W, Zemper ED (1997). Head and neck injuries in adult taekwondo athletes. Coaching Sport Sci J.

[12] Pieter W, Zemper ED (1998). Incidence of reported cerebral concussion in adult taekwondo athletes. J Royal Soci Promotion Health.

[13] Pieter W, Zemper ED (1999). Head and neck injuries in young taekwondo athletes. J Sports Med Phys Fitness.

[14] Beis K, Tsaklis P, Pieter W, Abatzides G (2001). Taekwondo competition injuries in Greek young and adult athletes. Eur J Sports Traumatol Rel Res.

[15] Koh JO, de Freitas T, Watkinson EJ (2001). Injuries at the 14th World Taekwondo Championships in 1999. Int J Applied Sports Sci.

[16] Koh JO, Watkinson EJ (2002). Possible concussions following head blows in the 2001 Canadian National Taekwondo Championships. Cross Boundaries- An Interdisciplinary J.

[17] Koh J. O, Watkinson E. J (2002). Video analysis of blows to the head and face at the 1999 World Taekwondo Championships. J Sports Med Phys Fitness.

[18] Kazemi M, Pieter W (2004). Injuries at a Canadian National Taekwondo Championships: a prospective study. BMC Musculoskeletal Disorders.

[19] Beis K, Pieter W, Abatzides G (2007). Taekwondo techniques and competition characteristics involved in time-loss injuries. J Sports Sci Med.

[20] Pieter W, Song JK, Yoo SH (2007). General and time-loss injuries in taekwondo. 1st International Symposium for Taekwondo Studies.

[21] Pieter W, Kazemi M, Song JK, Yoo SH (2007). Competition injuries in young Canadian taekwondo athletes. 1st International Symposium for Taekwondo Studies.

[22] Pieter W (2009). Cerebral concussions in adult taekwondo mechanisms and risk factors. The 2nd International Symposium for Taekwondo Studies.

[23] Lystad RP, Pollard H, Graham PL (2009). Epidemiology of injuries in competition taekwondo: A meta-analysis of observational studies. J Sci Med Sport.

[24] Ziaee V, Rahmani SH, Rostami M (2010). Injury rates in Iranian taekwondo athletes; a prospective study. Asian J Sports Med.

[25] Pieter W, Caine D, Harmer P, Schiff M (2009). Taekwondo. Epidemiology of Injury in Olympic Sports. International Olympic Committee Encyclopaedia of Sports Medicine.

[26] Pieter W, Zemper ED (1999). Injuries in adult American taekwondo athletes. Fifth IOC World Congress on Sport Sciences.

[27] Pieter W, Bercades LT, Heijmans J (1998). Injuries in young and adult taekwondo athletes. Kinesiology.

[28] Dick R, Ferrara MS, Agel J (2007). Descriptive epidemiology of collegiate men's football injuries: National Collegiate Athletic Association Injury Surveillance System, 1988–1989 through 2003–2004. J Athletic Training.

[29] Pieter W (2000). Injuries and mechanisms of injury in karate competition. 1st World Congress on Combat Sports and Martial Arts.

[30] Halabchi F, Ziaee V, Lotfian S (2007). Injury profile in women Shotokan Karate Championships in Iran (2004–2005). J Sports Sci Med.

[31] James G, Pieter W (2003). Injury rates in adult elite judoka. Biol Sport.

[32] Pieter W, Talbot C, Pinlac V, Bercades LT (2001). Injuries at the Konica Asian Judo Championships. Acta Kinesiologiae Universitatis Tartuensis.

[33] Ariazza R, Leyes M (2005). Injury profile in competitive karate: prospective analysis of three consecutive World Karate Championships. Knee Surg Sports Traumatol Arthrosc.

[34] Agel J, Ransone J, Dick R (2007). Descriptive epidemiology of collegiate men's wrestling injuries: National Collegiate Athletic Association Injury Surveillance System, 1988–1989 through 2003–2004. J Athletic Training.

[35] Zemper ED (1989). Injury rates in a national sample of college football teams: a 2-year prospective study. Phys Sports Med.

[36] Seward H, Orchard J, Hazard H, Collinson D (1993). Football injuries in Australia at the ćlite level. Med J Australia.

[37] Tuominen R (1995). Injuries in national karate competitions in Finland. Scand J Med Sci Sports.

[38] Beis K, Pieter W, Abatzides G, Jürimäe T, Jürimäe J (2001). Match characteristics and taekwondo injuries. Proceedings of the 7th International Scientific Conference of the International Association of Sport Kinetics. Acta Kinesiologiae Universitatis Tartuensis.

[39] Jakubiak N, Saunders DH (2008). The feasibility and efficacy of elastic resistance training for improving the velocity of the Olympic taekwondo turning kick. J Strength Cond Res.

[40] Wang HH, Liu C, Chu MY (2005). Effects of passive repeated plyometric training on specific kicking performance of elite Olympic taekwondo player (sic). ISB XXth Congress - ASB 29th Annual Meeting.

